# Supplementary material for: Gcm counteracts Toll-induced inflammation and impacts hemocyte number through cholinergic signaling
Source: Front Immunol. 2023 Nov 15;14:1293766. doi: 10.3389/fimmu.2023.1293766 (PMC10684909; doi:10.3389/fimmu.2023.1293766)
Supplement: Supplementary file 1 [file DataSheet_1.pdf]

Supplementary Figures, methods and Table

Gcm counteracts Toll-induced inflammation and impacts hemocyte number through cholinergic signaling

Authors:

Wael Bazzi 1,2,3,4
Sara Monticelli 1,2,3,4
Claude Delaporte 1,2,3,4
Céline Riet 1,2,3,4
Angela Giangrande 1,2,3,4,§
Pierre B. Cattenoz 1,2,3,4,§

Affiliations :
1. Université de Strasbourg, IGBMC UMR 7104- UMR-S 1258, F-67400 Illkirch, France
2. CNRS, UMR 7104, F-67400 Illkirch, France
3. Inserm, UMR-S 1258, F-67400 Illkirch, France
4. IGBMC, Institut de Génétique et de Biologie Moléculaire et Cellulaire, F-67400 Illkirch, France
§ corresponding authors

List of files :

Supplementary Figure S1: priming hemocytes enhance the melanotic tumors induced by Toll.

Supplementary Figure S2: Transcriptomic analysis of sensitized hemocytes in Toll10b background.

Supplementary Figure S3: nAchRalpha6 is expressed in resident hemocytes.

Supplementary Figure S4: Quantification of apoptotic hemocytes in nAchRalpha6 knock down and gain of function animals.

Supplementary methods

Supplementary Figure S1: priming hemocytes enhance the melanotic tumors induced by Toll.

A,B) Lymph glands from *w1118* (**A**) and *Toll*^{10b}*/+* (**B**) WL3 labelled with anti-L4 (lamellocyte marker in green), anti-Pxn (mature hemocyte marker in red) and DAPI. The lobes are indicated with white dashed lines.

C) Expression levels of the anti-microbial peptides AttB, AttA and CecB, of the cytokines Upd2 and Upd3 as well as of inhibitors and downstream targets of the Jak/Stat pathway in hemocytes *w1118* (in gray) and *gcm*^{26}*/+* (in blue) estimated by quantitative PCR. N = 4 pools of 10 larvae, the ANOVA indicated no significant modulation between the two genotypes.

Supplementary Figure S2: Transcriptomic analysis of sensitized hemocytes in *Toll*^{10b} background.

A) Outline of the pipeline followed to generate and analyse the transcriptome of hemocytes from *gcm*^{26}, *Toll*^{10b} and *gcm*^{26}*/+;Toll*^{10b}*/+* larvae. Three replicates were sequenced per genotype.

B) Number of reads and percentage of uniquely mapped reads for the 9 transcriptomes.
C) GO term enrichment analysis for genes down regulated or up regulated in *gcm*^{26}*/+;Toll*^{10b}*/+* compared to *gcm*^{26}. The size of the dots represents the enrichment and the color gradient from white to purple shows the significance of the enrichment, expressed in -Log10(p-value) with white being non-significant and blue highly significant.
D) Transcriptome comparison of hemocytes from WL3 *gcm*^{26} and *gcm*^{26}*/+;Toll*^{10b}*/+*. The x-axis represents the average genes expression levels (n=3) and the y-axis the Log2 Fold Change *gcm*^{26}*/+;Toll*^{10b}*/+/gcm*^{26}. The red dots highlight the genes of the Jak/Stat pathway.
E) GO term enrichment analysis for genes down regulated or up regulated in *gcm*^{26}*/+;Toll*^{10b}*/+* compared to *Toll*^{10b}. The dotplot is represented as in **(C)**. Note that the p-values are higher than in **C**.
F) Schematic representation of the Toll signaling pathway. The ligand Spätzle (Spz) binds to the receptor Toll (Tll) and induces the phosphorylation of the inhibitor of NF-κB Cactus (cact) through the interaction with the adaptor proteins Myd88 and Tube (Tub) and the tyrosine kinase Pelle (Pll). The phosphorylation of Cact induces its degradation, which release the NF-κB transcription factors Dorsal (Dl) and Dorsal-related immunity factor (Dif). Dl/Dif translocate to the nucleus and induce the expression of effector genes such as the antimicrobial peptides *Drosocin* (*Dro*), *Diptericin B* (*DptB*), *Attacin C* (*AttC*) or the serine protease *SPH93* (32).

Supplementary Figure S3: *nAchRalpha6* is expressed in resident hemocytes.

A-C''') Filet **(A-B'')** and hemocytes **(C-C''')** immunolabellings of L3 larvae *nAchRalpha6-T2A-Gal4/srp(hemo)3xmcherry;UAS-encGFP/+*. The field in **(A-B'')** focus on resident hemocytes located in the dorsal-posterior area of the larva. The hemocytes are labelled with anti-RFP (in red) and the cells expressing *nAchRalpha6-T2A-Gal4* with anti-GFP (in green). In **(A-B'')**, the gray channel represents anti-HRP labelling that shows neurons and in **(C-C''')** it represents anti-P1 labelling that show hemocytes. The complete stack projections are shown in **(A,C-C''')**, a single section is shown in **(B-B'')**.
D-F''') Immunolabelling of L3 filets expressing the fusion protein nAchRalpha6-YFP from the endogenous locus of *nAchRalpha6* (45). The YFP is labelled with anti-GFP (in green), the neurons with anti-HRP (in red) and the hemocytes with anti-He (in gray). A single section of a field covering resident hemocytes located in the dorsal-posterior part of the larva is shown in **(D-D''')** and full stack of a field located in the ventral part is shown in **(E-E''')**. **(F-F''')** show a single section at higher magnification of the hemocyte squared in **(E)**. Note the localisation of nAchRalpha6-YFP at the level of the cytoplasmic membrane as well as in cytoplasmic endosomes.
G-K) Filet immunolabellings reporting *nAchRalpha6* expression in L3 larvae Control (*nAchRalpha6-T2A-Gal4/UAS-nRFP*, **G-G'''**), *gcm*^{26}*/+* (*nAchRalpha6-T2A-Gal4/gcm*^{26}*,UAS-nRFP*, **H-H'''**), *Toll*^{10b}*/+*

(*nAchRalpha6-T2A-Gal4/UAS-nRFP; Toll^{10b}/+*, **I-I'''**) and *gcm^{26}/+;Toll^{10b}/+* (*nAchRalpha6-T2A-Gal4/gcm^{26},UAS-nRFP; Toll^{10b}/+*, **J-J'''**). The fields focus on resident hemocytes located in the dorsal-posterior area of the larva. The hemocytes are labelled with anti-Pxn (in gray) and anti-He (in green), the cells expressing *nAchRalpha6-T2A-Gal4* with anti-RFP (in red). The *nAchRalpha6* hemocytes are displayed in **(K)**, they were quantified on three filets per condition in more than 200 hemocytes over the three fields. The p-value was estimated by Chi² test.

In **(A-J''')**, the arrowheads indicate the hemocytes expressing *nAchRalpha6*.

Supplementary Figure S4: Quantification of apoptotic hemocytes in *nAchRalpha6* knock down and gain of function animals.

A-B") Immunolabelling of hemocytes from WL3 *HmldeltaGal4,Dbgtrace* with pH3 mitotic marker **(A-A'')** or with Dcp-1 apoptotic marker **(B-B")**. The hemocytes are labelled with anti-GFP (in green), anti-pH3 (in red) or anti-Dcp-1 (in grey) and the nuclei with DAPI (in blue). Scale bar: 20 µm.

C,D) Quantification of the apoptotic hemocytes in WL3 (120 hAEL) *HmldeltaGal4,Dbgtrace* and *HmldeltaGal4,Dbgtrace,nAchRalpha6-KD* **(A)** or *Pxn>nAchRalpha6 CRISPR GOF* and control **(D)**; n = 12, with more than 300 hemocytes scored for each replicate, p-values were estimated by one-factor ANOVA.

Supplementary Table S1: expression matrix from the RNAseq data on sensitized hemocytes in *Toll^{10b}* background.

Columns A,B: Gene Id, FBgn and Symbol.

Columns C-K: raw read counts for each sample (3 samples *gcm^{26}/+*, 3 samples *gcm^{26}/+;Toll^{10b}/+* and 3 samples *Toll^{10b}/+*.

Columns L-T: normalized read count from DEseq2 on each sample.

Column U: gene symbol

Column V: gene expression base mean across the whole dataset

Columns W-Y: mean expression in *gcm^{26}/+*, *gcm^{26}/+;Toll^{10b}/+* and *Toll^{10b}* hemocytes.

Columns Z-AC: Fold change, log2fold change, p-value and adjusted p-value estimated with DEseq2 for the comparison of *Toll^{10b}/+* and *gcm^{26}/+;Toll^{10b}/+* hemocytes.

Columns AD-AG: Fold change, log2fold change, p-value and adjusted p-value estimated with DEseq2 for the comparison of *gcm^{26}/+* and *gcm^{26}/+;Toll^{10b}/+* hemocytes.

Column AH: genes annotated as transmembrane receptors according to Flybase.

Supplementary Figure S1

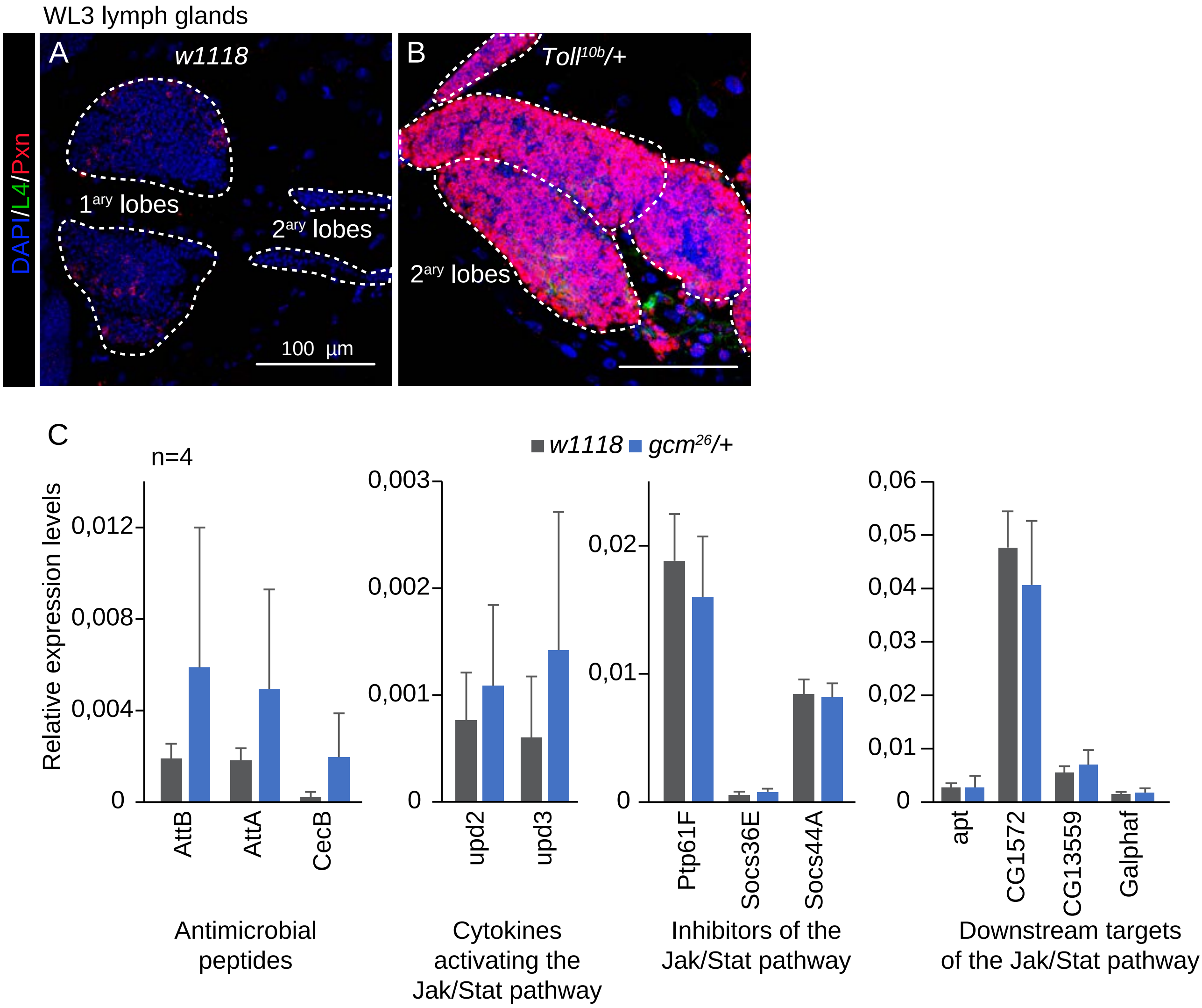

Supplementary Figure S2

A

Hemocytes from 100 larvae WL3 (n=3)	Transcriptome generation	Transcriptome analysis
gcm^{26}/+	Stranded mRNA sequencing	Mapping: Tophat (v1.4.0, genome dm6)
Toll^{10b}/+	(Illumina Hiseq 2500,	Expression levels: HTseq (v0.6.0)
gcm^{26}/+;Toll^{10B}/+	single end, 50nt)	Differential expression: DESeq2 (v0.99.2)

B

Genotype	Replicate	Sample_ID	Number of reads	% of reads uniquely mapped
gcm^{26}/+	1	WLBZ10 S1	33,745,068	78.45%
gcm^{26}/+	2	WLBZ11 S1	30,846,975	71.87%
gcm^{26}/+	3	WLBZ12 S1	33,718,526	76.17%
Toll^{10b}/+	1	WLBZ1 S1	51,491,906	86.29%
Toll^{10b}/+	2	WLBZ2 S1	48,991,987	86.35%
Toll^{10b}/+	3	WLBZ3 S1	50,968,016	86.18%
gcm^{26}/+;Toll^{10B}/+	1	WLBZ7 S2	62,296,451	88.30%
gcm^{26}/+;Toll^{10B}/+	2	WLBZ8 S2	46,443,939	86.64%
gcm^{26}/+;Toll^{10B}/+	3	WLBZ9 S2	60,029,803	87.87%

C

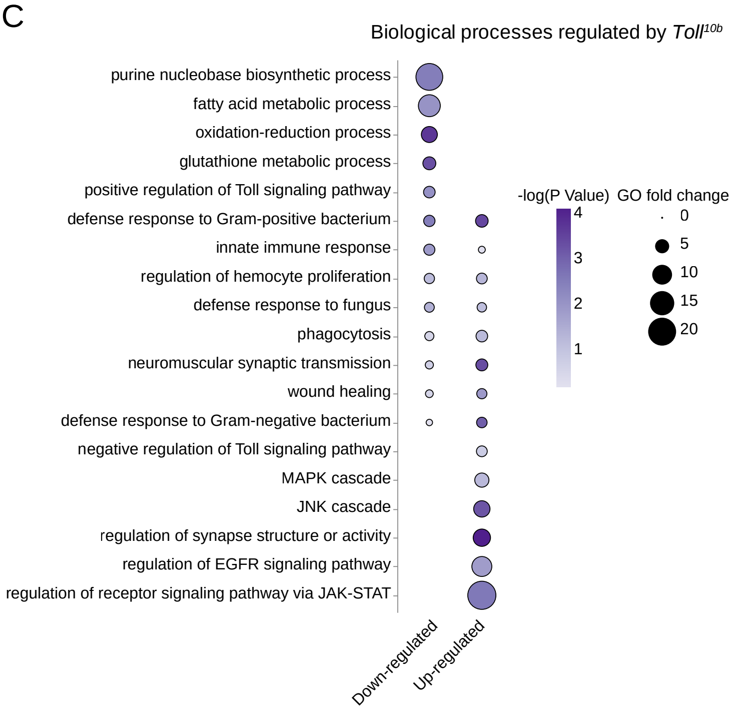

D

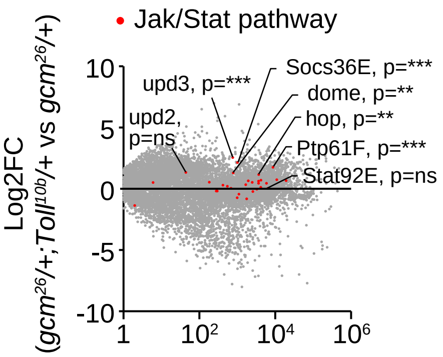

E

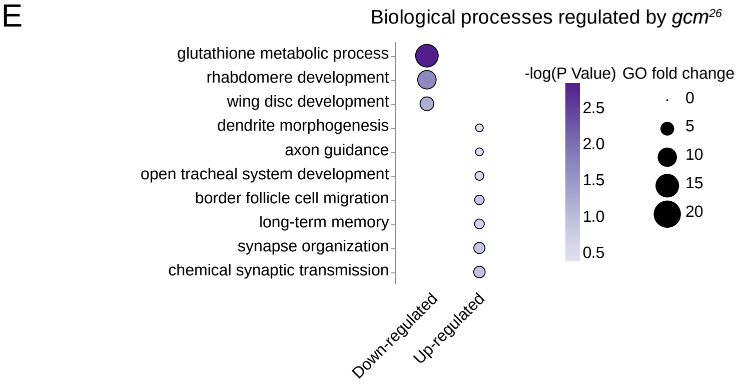

F

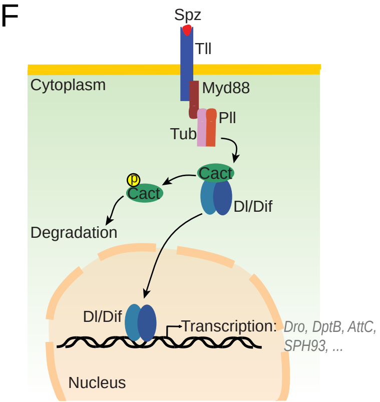

Supplementary Figure S3

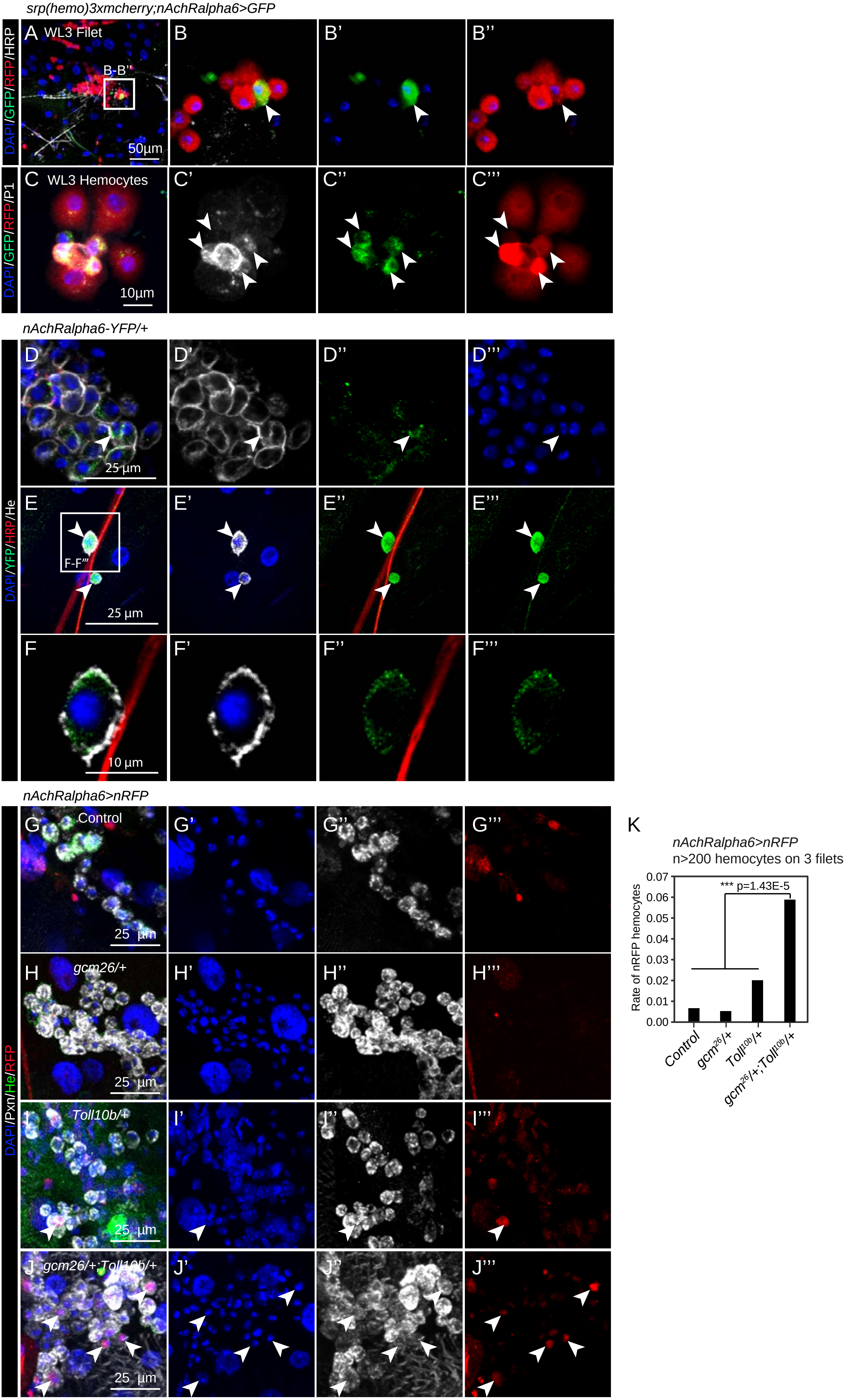

Supplementary Figure S4

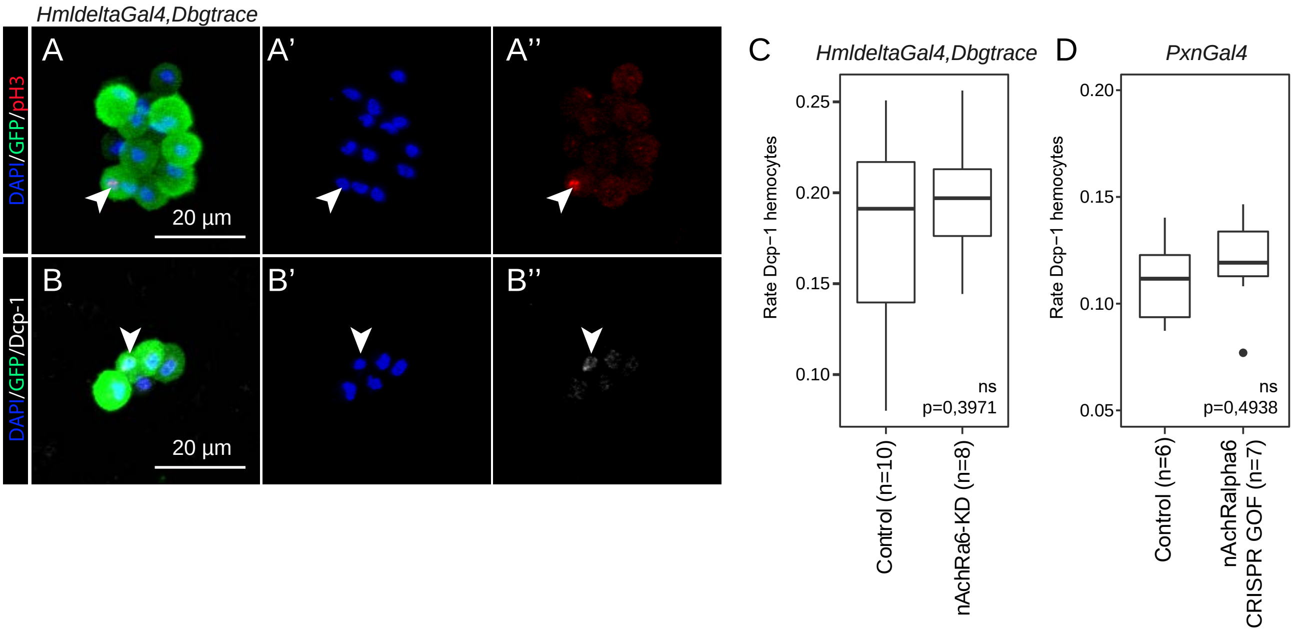

Supplementary methods

Fly strains and genetics

Flies were raised on standard media at 25°C. The following stocks were used:

Genotypes	Abbreviation	Origin	Remarks
gcmGal4/CyO,Tb	*gcm>*	(Soustelle and Giangrande 2007)	Driver specific to embryonic hemocytes and glia, *gcm* hypomorphic mutation
Toll^{10b}/TM3_Ser mwh[1] e[1] Tl[^{10b}]/T(1;3)OR60/TM3. Sb[1] Ser[1]	*Toll^{10b}/Ser, TM3*	(Schneider *et al.* 1991; Huang *et al.* 1997)	Constitutively activated Toll.
UAS-gcmRNAi	*gcm KD*	Bloomington #31519	dsRNA targeting *gcm* with *UAS* promoter
UAS-gcmF18A	*gcm GOF*	(Bernardoni *et al.* 1997)	*gcm* gain of function (GOF) under *UAS* promoter
gcm^{26}/CyOactinGFP	*gcm^{26}*	(Vincent *et al.* 1996)	Null *gcm* mutation
srp(hemo)Gal4	*srp(hemo)>*	(Bruckner *et al.* 2004)	Driver specific to embryonic hemocytes
Df(2L)132	*Df(2L)132*	(Kammerer and Giangrande 2001)	Chromosomal deletion of the locus covering *gcm* and *gcm2*.
srp(hemo)-3xmcherry	*Srp(hemo) 3xmcherry*	(Gyoergy *et al.* 2018)	Hemocyte reporter
w[*]; P(w[+mC]=UAS-GFP.S65T)eg[T10]	*UAS-GFP*	Bloomington #1522	*GFP* reporter under *UAS* promoter
nAchRbeta3-T2A-Gal4	*nAchRbeta3-T2A-Gal4*	(Kondo *et al.* 2020)	Driver reporting *nAchRbeta3* expression
nAchRalpha6-T2A-Gal4	*nAchRalpha6-T2A-Gal4*	(Kondo *et al.* 2020)	Driver reporting *nAchRalpha6* expression
Dop1R1-T2A-Gal4	*Dop1R1-T2A-Gal4*	(Kondo *et al.* 2020)	Driver reporting *Dop1R1* expression
w[1118]; P(w[+mC]=Hml-Gal4.Delta)2	*HmldeltaGal4*	Bloomington #30139	Driver specific to hemocytes
PxnGal4 (chr3)	*PxnGal4*	Gift from Pr. D. Hultmark	Driver specific to hemocytes
y[1]v[1];P(y[+t7.7]v[+t1.8]=TRiP.JF01851)attP2	*UAS-5HT1B-RNAi*	Bloomington #25833	dsRNA targeting *5-HT1B* with *UAS* promoter
y[1]v[1];P(y[+t7.7]v[+t1.8]=TRiP.HM04077)attP2	*UAS-Dop1R1-RNAi*	Bloomington #31765	dsRNA targeting *Dop1R1* with *UAS* promoter
y[1]v[1];P(y[+t7.7]v[+t1.8]=TRiP.JF03126)attP2	*UAS-Ekar-RNAi*	Bloomington #28506	dsRNA targeting *Ekar* with *UAS* promoter
y[1]v[1];P(y[+t7.7]v[+t1.8]=TRiP.JF01853)attP2	*UAS-nAchRalpha6-RNAi*	Bloomington #25835	dsRNA targeting *nAchRalpha6* with *UAS* promoter
UAS-nAchRbeta3-RNAi (chr2)	*UAS-nAchRbeta3-RNAi*	VDRC ID: KK 101868	dsRNA targeting *nAchRbeta3* with *UAS* promoter

y[1]v[1];P(y[+t7.7]v[+t1.8]=TRiP.HMS05847)attP40	UAS-RFP-RNAi	Bloomington #67984	dsRNA targeting *RFP* with *UAS* promoter (Control)
nAchRalpha6-YFP	*nAchRalpha6-YFP*	(KORONA *et al.* 2021)	Reporter of *nAchRalpha6*
nAChRalpha6[DAS1]	*nAChRα6*^{DAS1}	Bloomington #9685	Null mutation of *nAchRalpha6*. The genetic background was homogenised by chromosome replacement with the wild type stock Oregon-R
nAChRalpha6[DAS2]	*nAChRα6*^{DAS2}	Bloomington #9686	Null mutation of *nAchRalpha6*. The genetic background was homogenised by chromosome replacement with the wild type stock Oregon-R
w[*];P(w[+mC]=UAS-FLP.Exel)3,P(w[+mC]=Ubi-p63E(FRT.STOP)Stinger)15F2	*gtrace 3*	Bloomington #28282	Lineage tracing stock on chr 3
y,w,UAS-FLP;Act5C-FRT-y+-FRT-GAL4,UAS-GFP	*gtrace 2*	(HONTI *et al.* 2010)	Lineage tracing stock on chr X and 2
y,w,UAS-FLP;Act5C-FRT-y+-FRT-GAL4,UAS-GFP ;P(w[+mC]=UAS-FLP.Exel)3,P(w[+mC]=Ubi-p63E(FRT.STOP)Stinger)15F2	*Dbgtrace*	Build in this study	Stock carrying the two lineage tracing constructs *gtrace 2* and *gtrace 3*.
y[1]sc[*]v[1]sev[21];P(y[+t7.7]v[+t1.8]=TRiP.HMC05021)attP40	*UAS-ChAT-RNAi*	Bloomington #60028	dsRNA targeting *ChAT* under *UAS* promoter
y[1] w[*]; P(w[+m]=nSyb-GAL4.S)3*	*nSyb-Gal4*	Bloomington #51635	Neuronal *Gal4* driver
TI(2A-lexA::GAD)ChAT[2A-lexA]	*ChAT-T2A-LexA*	Bloomington #84379	Cholinergic neuron *LexA* driver
w[1118]; P(w[+mC]=ChAT-GAL4.7.4)19B/CyO, P(ry[+t7.2]=sevRas1.V12)FK1	*ChAT-Gal4*	Bloomington #6798	Cholinergic neuron *Gal4* driver
21-7-Gal4	*21-7-Gal4*	(HU *et al.* 2017)	Multidendritic neuron *Gal4* driver
y[1]w[*];P(w[+mW.hs]=GawB)109(2)80,P(w[+mC]=UAS-mCD8::GFP.L)LL5 (1;2)	*109(2)80-Gal4*	Bloomington #8768	Multidendritic neuron *Gal4* driver
P(w[+mW.hs]=GawB)OK6 (2)	*OK6-Gal4*	Bloomington #64199	Motorneuron *Gal4* driver
w; P(Tdc2-GAL4.C)2*	*Tdc2-Gal4*	Bloomington #9313	Octopaminergic neuron *Gal4* driver
w[1118]	*w1118*		Wild type
Oregon-R	*Oregon-R*		Wild type
w[1118]; P(y[+t7.7] w[+mC]=UAS-Cas9.C)attP2	*UAS-Cas9*	Bloomington #54595	

y[1] sc[*] v[1] sev[21]; P(y[+t7.7] v[+t1.8]=TKO.GS00614)attP40	*nAchRalpha6-TKO*	Bloomington # 76441	Expresses sgRNAs ubiquitously targeting *nAChRalpha6* for knock down with *Cas9*
y[1] w[*]; P(y[+t7.7] w[+mC]=UAS-3xFLAG.dCas9.VPR)attP40	*UAS-dCas9-VPR*	Bloomington #66561	
y[1] sc[*] v[1] sev[21]; P(y[+t7.7] v[+t1.8]=TOE.GS01969)attP40	*nAchRalpha6-TOE*	Bloomington #80510	Expresses sgRNAs ubiquitously targeting *nAChRalpha6* for overexpression with *dCas9-VPR*
nAchRalpha6-TKO ;PxnGal4		Build in this study	Crossed with *UAS-Cas9* to generate *nAchRalpha6 CRISPR LOF*
nAchRalpha6-TOE ;PxnGal4		Build in this study	Crossed with *UAS- dCas9-VPR* to generate *nAchRalpha6 CRISPR GOF*
nAchRalpha6-TKO/UAS-Cas9;PxnGal4/+	*nAchRalpha6 CRISPR LOF*	Build in this study	
UAS-Cas9/+;PxnGal4/+	*Control nAchRalpha6 CRISPR LOF*	Build in this study	
nAchRalpha6-TOE/UAS-dCas9-VPR;PxnGal4/+	*nAchRalpha6 CRISPR GOF*	Build in this study	
UAS- dCas9-VPR/+;PxnGal4/+	*Control nAchRalpha6 CRISPR GOF*	Build in this study	

Estimation of the penetrance of the melanotic tumors

Overnight lays of the parental crosses were incubated at 25°C until the wandering 3rd instar larval (WL3) stage. The larvae were then washed with water and tumor penetrance was determined by assessing the percentage of larvae carrying one or more tumors. More than 50 larvae were scored per genotype. The p-values were estimated using the chi-squared test for frequency comparisons.

Hemocyte counting

Parental crosses were allowed to lay eggs on collecting plates for 3hrs at 25°C. After 24hrs at 25°C, 100 1st instar larvae were transferred from the collecting plate to fresh vials (to normalise the number of animals per vial, avoid overcrowding and stage the larvae). At wandering stage (~116hrs after egg laying (hAEL)), 10 female larvae were washed and bled in PBS containing few crystals of N-phenylthiourea ≥98% (PTU) (Sigma-Aldrich P7629) to prevent hemocyte melanisation (LERNER AND FITZPATRICK 1950). During the bleeding procedure, the cuticle was scraped with forceps to recover the sessile hemocytes (PETRAKI *et al.* 2015). The hemocytes were then numerated on a Malassez counting cell and reported as number of hemocytes per larva. At least 3 replicates were performed for each genotype. The counts were compared using ANOVA followed by post hoc Tuckey HSD or bilateral student test as mentioned on each graph.

For the lamellocytes, 10 WL3 were treated as stated above and bled in PBS/PTU. The hemocytes were transferred onto a slide using the Cyto-Tek® 4325 Centrifuge (Miles Scientific). Samples were then fixed for 10min in 4% paraformaldehyde/PBS at room temperature (RT), incubated with blocking reagent (Roche) for 1hr at RT, incubated overnight at 4°C with primary antibodies targeting hemocyte (rabbit anti-Serpent, (TREBUCHET *et al.* 2019))and lamellocytes (Mouse anti-L4 kindly provided by I. Ando (HONTI

et al. 2010)) diluted in blocking reagent. The slides were then washed three times for 10min with PTX (PBS, 0.3% triton-x100), incubated for 1hr with secondary antibodies, washed twice for 10min with PTX, incubated for 20min with DAPI to label nuclei (Sigma-Aldrich) (diluted to 10^{-3} g/L in blocking reagent), and then mounted in Vectashield® (Vector Laboratories). The slides were analysed by confocal microscopy. Each immunolabelling was carried out on three independent trials. The p-values were estimated after variance analysis using bilateral student test.

Lymph gland, filet and whole larva immunolabelling

Lymph glands of the indicated stages were dissected in Ringer's solution (pH 7.3-7.4), fixed for 30min in 4% PFA/PBS at 4°C, washed three times for 10min with PTX, incubated with blocking reagent for 1hr at 4°C, incubated overnight at 4°C with primary antibodies, washed three times for 10min with PTX, incubated for 1hr with secondary antibodies, washed two times for 10min with PTX, incubated for 20min with DAPI and then mounted on slides in Vectashield®. The slides were analysed by confocal microscopy.

The filets were prepared in cold PBS following the protocol from Brent et al. with modifications (BRENT *et al.* 2009). Briefly, to expose resident hemocytes located in dorsal and lateral positions, the larvae were dissected ventrally or laterally on a cold block to prevent dislodging the resident hemocytes. The organs were removed gently from the pinned larvae. Then the filets were fixed in 4% PFA in 1x PBS for 30min and treated as described above for the lymph glands.

Whole larva labelling was carried out following the protocol from (MANNING AND DOE 2017) with modifications. Briefly, the feeding L3 were collected and washed with water, treated 10min with 100 % bleach and rinsed in water. Then the larvae were treated with proteinases for 1h at 37°C in CCD buffer (chitinase (5 U/ml), chymotrypsin (100 U/ml) in HEPES buffer (25 mM) with 1,5% (vol/vol) DMSO) supplemented with 7 M urea to improve antibody penetration. The larvae were then fixed in 9% (vol/vol) PFA in PBS with heptane for 30 min at room temperature and treated with heptane and methanol (cracking procedure). A second fixation was carried out for 7 days at -20°C with 2% PFA in methanol. Heptane was added to increase the permeability of the animals for the immunostaining. Following this, the larvae were treated with Proteinase K to increases the coloration in deep tissue, blocked overnight with blocking reagent (Roche) in PTX, incubated 7 days with the primary antibodies and 1 day with the secondary antibodies.

For all the immunolabellings, the following primary antibodies were used: mouse anti-Hemese (1/30), (KURUCZ *et al.* 2003) and mouse anti-P1 (1/30), kindly provided by I. Ando (KURUCZ *et al.* 2007); rabbit anti-PH3 (1/1000) (Upstate biotechnology #06-570); chicken anti-GFP (1/500) (abcam #13970); rat anti-RFP (1/500) (Chromotek #5F8-100); rabbit anti-HRP (1/500) to label neurons (JAN AND JAN 1982); mouse anti-Dlg (DSHB Cat# 4F3 anti-discs large, RRID:AB_528203); mouse anti-Brp (DSHB Cat# nc82, RRID:AB_2314866) and the following secondary antibodies: Cy3 Donkey Anti-Mouse IgG (Jackson ImmunoResearch Labs Cat# 715-165-151, RRID:AB_2315777); Cy3 Goat Anti-Rat IgG (Jackson ImmunoResearch Labs Cat# 112-165-167, RRID:AB_2338251); Cy5 Goat Anti-Mouse IgG (Jackson ImmunoResearch Labs Cat# 115-177-003, RRID:AB_2338719); Cy5-AffiniPure Goat Anti-Rat IgG (H+L) (Jackson ImmunoResearch Labs Cat# 112-175-167, RRID:AB_2338264) and FITC anti-chicken (Jackson ImmunoResearch Labs Cat#703-095-155).

Confocal imaging

Leica SP8 inverted-based microscope equipped with 20, 40 and 63X objectives was used to obtain confocal images. GFP/FITC was excited at 488nm; the emission filters 498-551 were used to collect the signal. Cy3 was excited at 568nm; emission filters 648-701 were used to collect the signal, and Cy5 was excited at 633nm; emission signal was collected at 729-800nm. A step size between 0.2 and 2µm was used to collect the Z-series of images, which were then treated with Fiji (SCHINDELIN *et al.* 2012). For each set of images, the intensity of the signals was set to the same threshold in order to compare the different genotypes.

Stranded RNA sequencing on hemocytes from WL3 larvae

For each replicate, the hemocytes were bled from 100 WL3 of the following genotypes: (1) *gcm^{26}/+* issued from the cross *gcm^{26}/CyOGFP* x *w^{1118}*, (2) *Toll^{10b}/+* issued from the cross *Toll^{10b}/Ser,TM3* x *w^{1118}* and (3) *gcm^{26}/+;Toll^{10b}/+* issued from the cross *gcm^{26}/CyOGFP* x *Toll^{10b}/Ser,TM3*. The RNA were extracted using Tri reagent (Sigma) following the manufacturer's instruction. Library preparation and sequencing were carried out by the genomeast facility (https://www.igbmc.fr/plateformes-technologiques/genomeast) at the IGBMC. Stranded mRNA libraries were prepared with Directional mRNA-Seq (SamplePrep). The libraries were sequenced on the Illumina Hiseq 2500 as single-end 50 base reads following Illumina's instructions. Image analysis and base calling were performed using RTA 1.18.61 and CASAVA 1.8.2. The reads were mapped to the *Drosophila* genome dm6 using Tophat (v1.4.0) (TRAPNELL *et al.* 2009) with the following parameters: library type FR second strand, anchor length = 8, minimum intron length = 70, maximum intron length = 500000. The bam files were then converted to count files using Htseq count (ANDERS *et al.* 2015) on stranded data and compared using DESeq2 (VARET *et al.* 2016). The graphical representations were built using R version 4.1.0, R studio version 1.4.1717 and the packages ggplot2 (WICKHAM 2016) and pheatmap (RRID:SCR_016418).

The RNA-seq data have been deposited in the ArrayExpress database at EMBL-EBI (www.ebi.ac.uk/arrayexpress) under accession number ***E-MTAB-11970 (Reviewers login (view only): Username: Reviewer_E-MTAB-11970; Password: vomd2sqk).***

GO term enrichment analysis.

GO term enrichment analysis was done using PANGEA for multiple lists (HU *et al.* 2023) using the EXP GO Biological Processes database. Genes presenting a base mean expression above 100, an upregulation above 2 fold (Log2 fold change > 1) and an adjusted p-value below 0.01 were selected for the analysis.

RNA extraction and qPCR

For the qPCR validation of the transcriptomic data, 20 WL3 of the indicated genotypes were bled on ice cold PBS. The cells were then centrifuged at 1200 rpm at 4°C and RNA isolation was performed with Tri-reagent (Sigma) following the manufacture's protocol. The DNase treatment was done with the TURBO DNA-free kit (Invitrogen) and the reverse transcription (RT) with the Super-Script IV (Invitrogen) using random primers. The qPCR assays were done with FastStart Essential DNA Green Master (Roche) with the primers are listed below.

Target	Forward primer	Reverse primer
AttA	GGCCCATGCCAATTTATTCA	AGCAAAGACCTTGGCATCCA
CecA2	CATTCTGGCCATCACCATTGGACA	GTGTGCTGACCAACACGTTCGATT
CecB	TTCGTCTTTGTGGCACTCATCCTG	GGTATGCTGACCAATGCGTTCGAT
GstE7	CGATGATTCCCAAGGAGCGT	GGTCGTGTCCACCTTTACGA
GstE1	CCGTACACCAGGGTCTGAAG	ATTGAGGCGATCCAACCAGG
nAchRalpha6	AGAGCCACGCGATACAAACA	GGTGGACAGCAGATGGTTCA
Rp49	GACGCTTCAAGGGACAGTATCTG	AAACGCGGTTCTGCATGAG
Act5C	GCAGCAACTTCTTCGTCACA	CTTAGCTCAGCCTCGCCACT

The expression levels were calculated relative to the two housekeeping genes Rp49 and Act5C levels using the ΔCt formula: $2^{(average(Ct_{Rp49}, Ct_{Act5c}) - Ct_{target})}$. Triplicate were done for each genotype and the levels were compared using bilateral student test after variance analysis.

DHE, pH3 and Dcp-1 quantification

For the estimation of the ROS levels, single *Toll^{10b}/+* and *gcm^{26}/+;Toll^{10b}/+* WL3 were bled in 20 µL of Schneider medium containing PTU on a 12 wells chamber slide (1 larva per well). The hemocytes were

left to decant for 20min and incubated in Schneider medium containing 10 µM dihydroethidium (DHE, Sigma) and Hoechst for 30min at RT in a wet chamber. The endogenous ROS convert the DHE to the fluorescent 2-hydroxyethidium (ZHAO *et al.* 2005), which absorbs 488nm light and emits photons at ~600nm. Hoechst labels the nuclei of the hemocytes. Following this, the medium was replaced by fresh Schneider medium and the slides were scanned using CellInsight CX7 instrument (Cellomics) at 20x magnification (25 fields per well). The intensity of the DHE within the nuclei delimited by the Hoechst signal was estimated using HCS Studio software and Colocalisation Bioapplication, for at least 7 larvae per genotype with more than 200 cells per larva. The DHE intensity averages were compared using bilateral student test.

For the estimation of the number of mitotic and apoptotic hemocytes, single WL3 were bled in 20 µL of Schneider medium containing PTU on a 12 wells chamber slide (1 larva per well). The hemocytes were left to decant for 20 min, fixed for 10min with 4% PFA in 1x PBS, washed with PTX, incubated for at least 1hr with blocking reagent and labelled with primary antibodies targeting the mitotic marker pH3 or the apoptotic marker cleaved Dcp-1 and secondary antibody coupled with Cy5, as well as DAPI to label the nuclei. The images were acquired using CellInsight CX7 instrument (Cellomics) at 20x magnification (100 fields per well). The image analysis was done using HCS Studio software and Colocalisation Bioapplication. The region of interest was defined using DAPI labelling and the pH3 or Dcp-1 intensity was measured for each region. The cells displaying a pH3 intensity above 6 times the median intensity or a Dcp-1 intensity above 1.8 times the median intensity of the well (determined empirically over 3 wells, **Supplementary Figure S4A,B**) were considered as proliferative for pH3 or apoptotic for Dcp-1. The rates of proliferation were compared using ANOVA.

Supplementary bibliography:

Anders, S., P. T. Pyl and W. Huber, 2015 HTSeq--a Python framework to work with high-throughput sequencing data. Bioinformatics 31**:** 166-169.

Bernardoni, R., V. Vivancos and A. Giangrande, 1997 glide/gcm is expressed and required in the scavenger cell lineage. Developmental Biology 191**:** 118-130.

Brent, J., K. Werner and B. D. McCabe, 2009 Drosophila larval NMJ immunohistochemistry. J Vis Exp.

Bruckner, K., L. Kockel, P. Duchek, C. M. Luque, P. Rorth *et al.*, 2004 The PDGF/VEGF receptor controls blood cell survival in Drosophila. Dev Cell 7**:** 73-84.

Gyoergy, A., M. Roblek, A. Ratheesh, K. Valoskova, V. Belyaeva *et al.*, 2018 Tools Allowing Independent Visualization and Genetic Manipulation of Drosophila melanogaster Macrophages and Surrounding Tissues. G3 (Bethesda) 8**:** 845-857.

Honti, V., G. Csordas, R. Markus, E. Kurucz, F. Jankovics *et al.*, 2010 Cell lineage tracing reveals the plasticity of the hemocyte lineages and of the hematopoietic compartments in Drosophila melanogaster. Mol Immunol 47**:** 1997-2004.

Hu, C., M. Petersen, N. Hoyer, B. Spitzweck, F. Tenedini *et al.*, 2017 Sensory integration and neuromodulatory feedback facilitate Drosophila mechanonociceptive behavior. Nat Neurosci 20**:** 1085-1095.

Hu, Y., A. Comjean, H. Attrill, G. Antonazzo, J. Thurmond *et al.*, 2023 PANGEA: a new gene set enrichment tool for Drosophila and common research organisms. Nucleic Acids Res 51**:** W419-W426.

Huang, A. M., J. Rusch and M. Levine, 1997 An anteroposterior Dorsal gradient in the Drosophila embryo. Genes Dev 11**:** 1963-1973.

Jan, L. Y., and Y. N. Jan, 1982 Antibodies to horseradish peroxidase as specific neuronal markers in Drosophila and in grasshopper embryos. Proc Natl Acad Sci U S A 79**:** 2700-2704.

Kammerer, M., and A. Giangrande, 2001 Glide2, a second glial promoting factor in Drosophila melanogaster. EMBO J 20**:** 4664-4673.

Kondo, S., T. Takahashi, N. Yamagata, Y. Imanishi, H. Katow *et al.*, 2020 Neurochemical Organization of the Drosophila Brain Visualized by Endogenously Tagged Neurotransmitter Receptors. Cell Rep 30**:** 284-297 e285.

Korona, D., B. Dirnberger, C. N. G. Giachello, R. M. L. Queiroz, D.-P. Minde *et al.*, 2021 Drosophila nicotinic acetylcholine receptor subunits and their native interactions with insecticidal peptide toxins. bioRxiv**:** 2021.2008.2013.456240.

Kurucz, E., R. Markus, J. Zsamboki, K. Folkl-Medzihradszky, Z. Darula *et al.*, 2007 Nimrod, a putative phagocytosis receptor with EGF repeats in Drosophila plasmatocytes. Curr Biol 17**:** 649-654.

Kurucz, E., C. J. Zettervall, R. Sinka, P. Vilmos, A. Pivarcsi *et al.*, 2003 Hemese, a hemocyte-specific transmembrane protein, affects the cellular immune response in Drosophila. Proc Natl Acad Sci U S A 100**:** 2622-2627.

Lerner, A. B., and T. B. Fitzpatrick, 1950 Biochemistry of melanin formation. Physiol Rev 30**:** 91-126.

Manning, L., and C. Q. Doe, 2017 Immunofluorescent antibody staining of intact Drosophila larvae. Nat Protoc 12**:** 1-14.

Petraki, S., B. Alexander and K. Bruckner, 2015 Assaying Blood Cell Populations of the Drosophila melanogaster Larva. J Vis Exp.

Schindelin, J., I. Arganda-Carreras, E. Frise, V. Kaynig, M. Longair *et al.*, 2012 Fiji: an open-source platform for biological-image analysis. Nat Methods 9**:** 676-682.

Schneider, D. S., K. L. Hudson, T. Y. Lin and K. V. Anderson, 1991 Dominant and recessive mutations define functional domains of Toll, a transmembrane protein required for dorsal-ventral polarity in the Drosophila embryo. Genes Dev 5**:** 797-807.

Soustelle, L., and A. Giangrande, 2007 Novel gcm-dependent lineages in the postembryonic nervous system of Drosophila melanogaster. Dev Dyn 236**:** 2101-2108.

Trapnell, C., L. Pachter and S. L. Salzberg, 2009 TopHat: discovering splice junctions with RNA-Seq. Bioinformatics 25**:** 1105-1111.

Trebuchet, G., P. B. Cattenoz, J. Zsamboki, D. Mazaud, D. E. Siekhaus *et al.*, 2019 The Repo Homeodomain Transcription Factor Suppresses Hematopoiesis in Drosophila and Preserves the Glial Fate. J Neurosci 39**:** 238-255.

Varet, H., L. Brillet-Gueguen, J. Y. Coppee and M. A. Dillies, 2016 SARTools: A DESeq2-and EdgeR-Based R Pipeline for Comprehensive Differential Analysis of RNA-Seq Data. Plos One 11.

Vincent, S., J. L. Vonesch and A. Giangrande, 1996 Glide directs glial fate commitment and cell fate switch between neurones and glia. Development 122**:** 131-139.

Wickham, H., 2016 ggplot2 : Elegant Graphics for Data Analysis, pp. 1 online resource (XVI, 260 pages 232 illustrations, 140 illustrations in color in *Use R!*,. Springer International Publishing : Imprint: Springer,, Cham.

Zhao, H., J. Joseph, H. M. Fales, E. A. Sokoloski, R. L. Levine *et al.*, 2005 Detection and characterization of the product of hydroethidine and intracellular superoxide by HPLC and limitations of fluorescence. Proc Natl Acad Sci U S A 102**:** 5727-5732.
